# Growth of epiphysis after epiphyseal-preservation surgery for childhood osteosarcoma around the knee joint

**DOI:** 10.1186/s12891-018-2109-4

**Published:** 2018-06-06

**Authors:** Akihiko Takeuchi, Norio Yamamoto, Katsuhiro Hayashi, Hidenori Matsubara, Hiroaki Kimura, Shinji Miwa, Takashi Higuchi, Kensaku Abe, Yuta Taniguchi, Hiroyuki Tsuchiya

**Affiliations:** Department of Orthopaedic Surgery, Kanazawa University Graduate School of Medical Sciences, 13-1 Takara-machi, Kanazawa, 920-8641 Japan

**Keywords:** Pediatric osteosarcoma, Epiphysis growth, Epiphyseal-preservation surgery

## Abstract

**Background:**

Epiphyseal-preservation surgery for osteosarcoma is an alternative method which has been indicated carefully to selected patients. The tumor-devitalised autograft treated with liquid nitrogen procedure is one of the biological reconstruction method to reconstruct the defect after tumor excision. The limb length discrepancy is usually appeared in children with their growth after limb-sparing surgery. This study was aimed to investigated the growth of residual epiphysis following epiphyseal-preservation surgery for childhood osteosarcoma around the knee joint.

**Methods:**

We retrospectively reviewed 12 patients with osteosarcoma who underwent epiphysis preserving tumor excision (8 in distal femur and 4 in proximal tibia) and reconstructed by using tumor-devitalized autograft treated with liquid nitrogen. The mean patient age was 11 (range, 6 to 14) years. The mean follow-up period were 63 (range, 41 to 90) months. Epiphysis transverse growth rate, epiphysis-width discrepancy (EWD) and collapse of epiphysis were evaluated by using pre- and post-operative whole standing leg radiographs. A retrospective chart review was performed to investigate functional outcome, complications and oncological status.

**Results:**

The mean growth of epiphysis rate was 12.6% (range, 3.3 to 28.0%) of affected side and 12.7% (range, 3.8 to 28.9%) of contralateral side, mean EWD was 0.1 mm (range, − 1.0 to 1.7 mm), mean LLD was + 26.1 mm (range, + 1 to + 48 mm) and two patients with distal femoral reconstruction underwent limb lengthening of tibia. There was no collapse of the residual epiphysis. The mean MSTS score was 27.7 (range, 18 to 30).

**Conclusions:**

Epiphysis transverse growth was not diminished, and there was absence of epiphyseal collapse even after epiphyseal-preservation surgery in this small series of childhood osteosarcoma around the knee. With careful assessment for epiphyseal tumor involvement, epiphyseal-preservation surgery shall be possible, and could be an alternative method worth considering.

## Background

The advances in imaging modalities, multi-agent chemotherapy and surgical procedures have made limb-sparing surgery more common in the treatment of osteosarcoma, which is the most common malignant bone tumor [1]. However, osteosarcoma usually occurs in metaphyseal locations and must be excised with the adjacent joint and replaced by an endoprosthesis. Moreover, treatment of skeletally immature children should consider any growth-related complications following tumor surgery. Limb-length discrepancy is a major complication [2], and extendable endoprostheses [3] or distraction osteogenesis [4] have been applied to address this problem.

Recently, the advanced imaging [5], accurate tumor excision [6] and rigid fixation using a locking plate [7] have made it possible to perform the epiphyseal-preservation surgery for selected patients who responded to chemotherapy without tumor extension to the epiphysis [8]. This procedure is expected to preserve excellent limb function. To reconstruct the defect after tumor excision, various methods, including allograft [8], distraction osteogenesis [9], tumor-devitalised autograft [10], vascularised fibular graft [11] and custom-made implants [6], have been applied. Tumor-devitalised autograft treated with liquid nitrogen procedure was introduced in 1999 [10] and its usefulness has since been reported [12]. The advantages of frozen autografts include simplicity and the possibility of preserving proteins, including bone morphogenetic protein (BMP) [13]. However, the growth of residual epiphysis in children after epiphyseal-preservation surgery has not been fully studied. The purpose of this study was to investigate the growth of residual epiphysis, limb function, complications and oncological status after epiphyseal-preservation surgery for childhood osteosarcoma around the knee.

## Methods

**Fig. 1:**
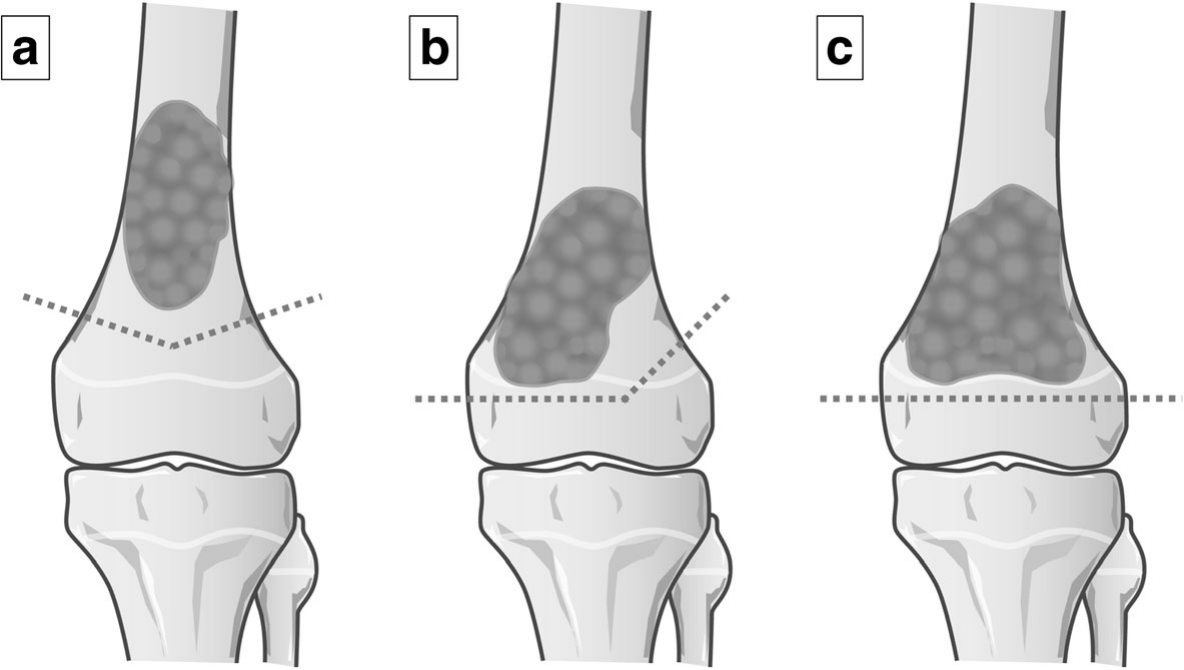
Diagram showing the classification of tumor excision to preserve the epiphysis. Transmetaphyseal excision (epiphyseal plate preserved) (**a**), transphyseal excision (epiphyseal plate partially sacrificed) (**b**) and transepiphyseal excision (epiphyseal plate totally sacrificed) (**c**). The dotted line indicates the osteotomy line

After the approval of our institutional review board, the authors retrospectively reviewed consecutive cases of osteosarcoma in our hospital treated with epiphysis preserving tumor excision and reconstion by using the tumor-devitalised autograft treated with liquid nitrogen procedure at our hospital between 2009 and 2013. All patients received neoadjuvant chemotherapy following our base protocol of five pre-operative courses of intra-arterial or intra-venous cisplatin (120 mg/m^2^) and doxorubicin (30 mg/m^2^/day × 2 days) [14]. The indications for this procedure were: 1) good radiological responses to neoadjuvant chemotherapy without extend to the physis, described as findings of sclerotic changes or good margination of the tumor observed on plain radiographs, marked shrinkage of tumors extending into soft tissue on MR images, or the disappearance of abnormal accumulation on TI 201 scintigrams [15]; 2) a thickness of their residual epiphysis > 1 cm; 3) a surgical margin of ≥10 mm. When tumor was located within 2 cm of the physis, the osteotomy line was transepiphyseal or passed through the physis, resulted in complete or partial ablation of the physis [16], and 4) either the absence of (Enneking stages IIA and IIB), or a resectable lung metastasis (Enneking stage III) [17]. The inclusion criteria of the present study was patients < 15 years old with open epiphyseal plate, follow-up of > 24 months and who had a tumor located in the distal femur or the proximal tibia. The patients with a diaphyseal osteosarcoma for which the distance of the osteotomy to the epiphyseal plate was < 1 cm were also included. The tumor excision was classified into three types: transmetaphyseal excision (epiphyseal plate preserved), transphyseal excision (epiphyseal plate partially sacrificed) and transepiphyseal excision (epiphyseal plate totally sacrificed) (Fig. 1). Transphyseal and transepiphyseal excision were indicated for tumors that did not extend to the epiphysis [16], as determined by magnetic resonance imaging (MRI) T1-weighted and short tau inversion recovery (STIR) images [5].

### Surgical procedure

Two different techniques of tumor-devitalised autograft treated with liquid nitrogen—free freezing [10] and pedicle freezing [18]—were used depending on the location of the tumor.

### Free freezing technique

**Fig. 2:**
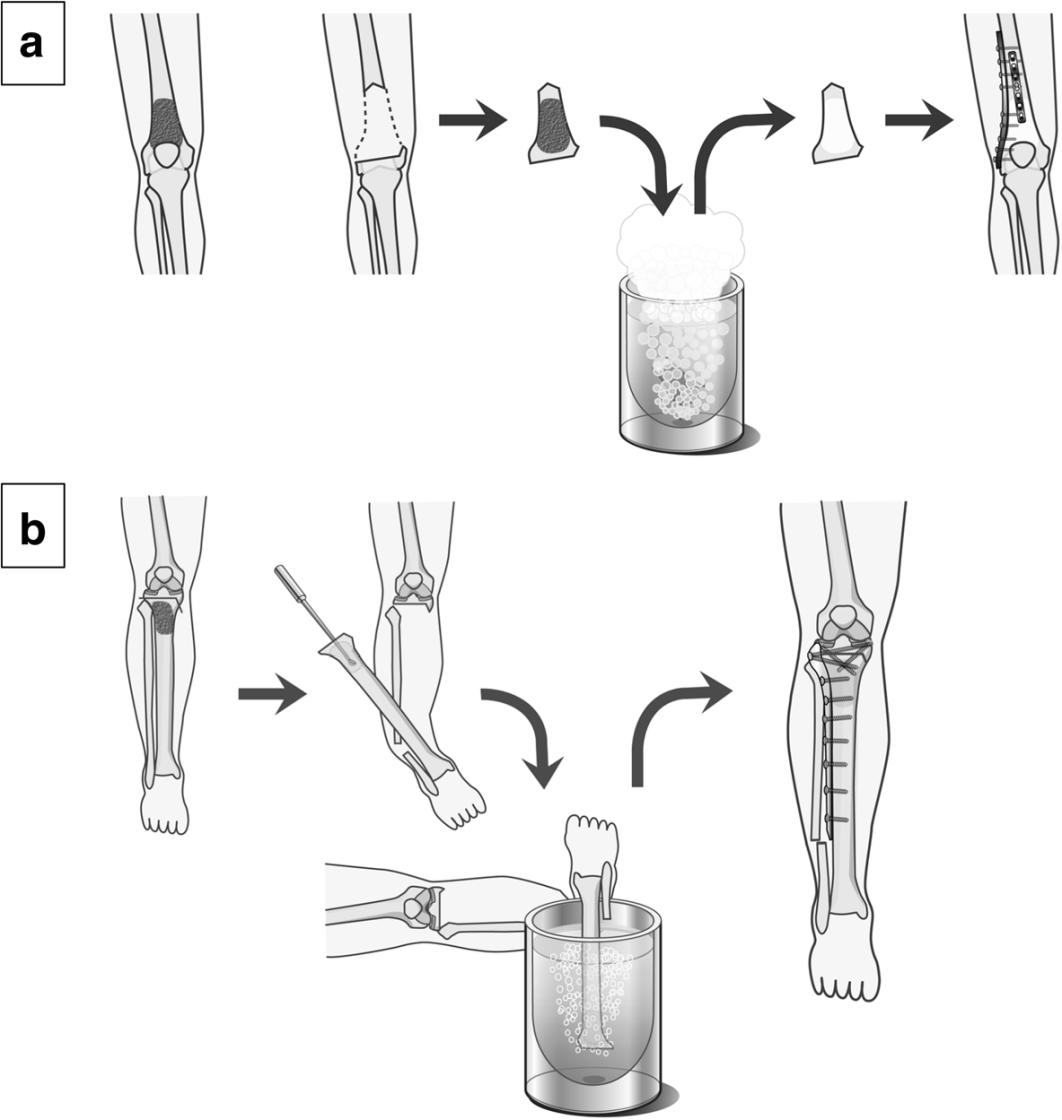
Schema of surgical procedure of tumor-devitalised autograft treated with liquid nitrogen. Free freezing: Tumor of the distal femur is excised by epiphysis-preserved intercalary osteotomy. After freezing in liquid nitrogen for 20 min, osteosynthesis using plates, screws is performed (**a**). Pedicle freezing: Tumor of the proximal tibia is excised by one- site osteotomy, proximal part of tumor is elevated and the intramedullary canal which contains the tumor, is subsequently curetted. The isolated bony specimen is carefully rotated and put in a liquid nitrogen for 20 min. Osteosynthesis using plate, screws is performed (**b**)

A K-wire was inserted into the osteotomy line under fluoroscopy. The tumor was then excised en bloc using a microsurgical saw along the K-wire. The specimen’s soft tissues were removed, and the tumor curetted before freezing. The excised portion was frozen in liquid nitrogen that was stored in sterilized flask right before freezing for 20 min, thawed at room temperature for 15 min, then in distilled water for another 15 min. The frozen autograft was then fixed to the residual limb with double or triple locking plates, and the epiphysis stabilized with two or three screws applied either through the plate or separately (Fig. 2a). For cases of proximal tibial osteosarcoma, the patella tendon was reattached to the frozen autograft by using a screw and spike washer.

### Pedicle freezing technique

After the osteotomy of the proximal side of the tumor with adequate surgical margin, the surrounding soft tissues were carefully protected by using surgical sheets and the proximal part of tumor was elevated with isolation by using cotton for cast padding, an Esmarch bandage, and three-layer surgical sheets to prevent tumor contamination and damages to the normal tissue during freezing. The intramedullary canal is subsequently curetted to remove the bone marrow and contents of the tumor in order to prevent a graft-related fracture due to water expansion during freezing. The isolated bony specimen, still in continuity with the distal residual limb, was then carefully rotated and put in a container filled with liquid nitrogen for 20 min. After thawing, reconstruction was performed similarly as for the free freezing technique (Fig. 2b).

The length of resection for free freezing, or of the treated bone for pedicle freezing, was recorded as the length of graft. Additional chemotherapy was administered during the postoperative period. Passive range of motion (ROM) therapy was initiated after the early postoperative period, and active ROM therapy was initiated at 6 weeks postoperatively when the muscle or tendon had to be reattached. The authors encouraged full weight-bearing mobilization after evidence of union. The follow-up protocol consisted of whole standing leg radiographs after 1.5, 3, 6, 9, 12, 16, 20 and 24 months and then every 6 months up to 5 years. Yearly examination was performed up to 10 years thereafter. Graft union was confirmed when the osteotomy line had disappeared in both anteroposterior (AP) and lateral views on radiography or computed tomography. When it was difficult to evaluate bone union because of the implant shadow, confirmation was achieved by using only one view (either AP or lateral) or computed tomographic scan. Graft union was not evaluated for cases of pedicle freezing for distal femoral tumors because the osteotomy line was only on the proximal side.

**Fig. 3:**
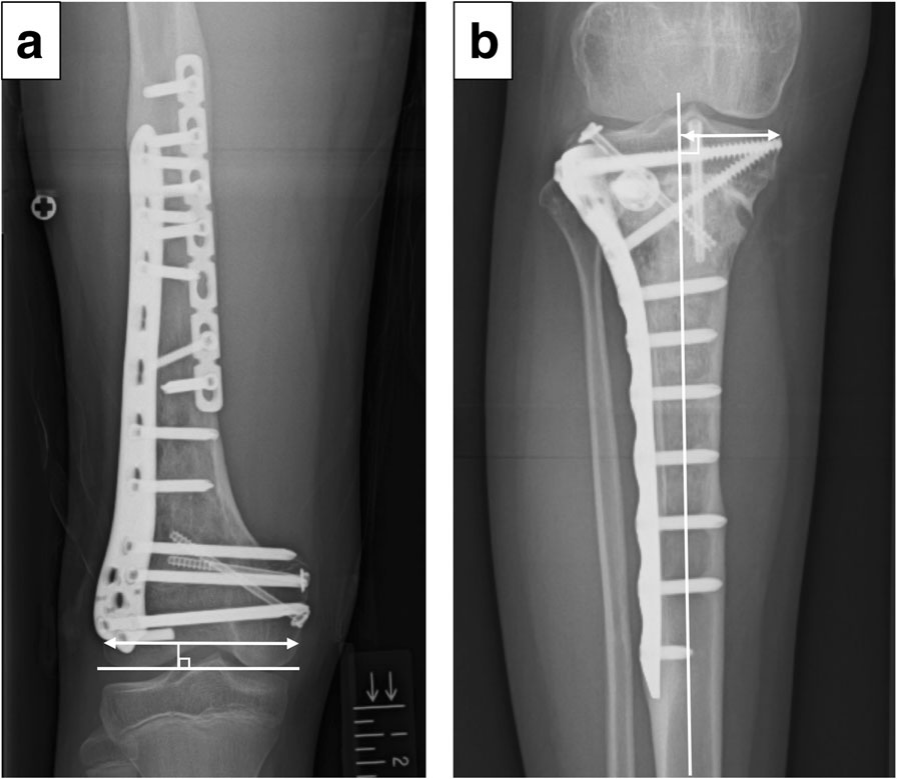
The width of epiphysis was measured by using whole standing leg radiographs with a 1-mm precision scale. The distance between the intersection of the line that was placed parallel to the joint surface and passed through the medial and lateral cortex at the level of intercondylar fossa in the distal femur before surgery and at the latest examination (a 10-year-old boy with osteosarcoma of the right distal femur, 55 months after surgery) (**a**). The width of the epiphysis of the proximal tibia was measured by a line that was perpendicular to the tibial anatomical axis and passes through the medial edge of the medial condyle. The distance between the ends of the medial and the cross point to the mechanical axis was measured on the proximal tibia before surgery and at the latest examination (an 11-year-old girl with osteosarcoma of the right proximal tibia, 42 months after surgery) (**b**)

The width of the epiphysis of the distal femur was measured with reference to the method of Souryal TO et al. [19]. The distance between the intersection of a line parallel to the joint surface and passing through the medial and lateral cortex at the level of the intercondylar fossa was measured by using whole standing leg radiographs with a 1-mm precision scale. The width of the epiphysis of the proximal tibia was measured from a line perpendicular to the tibial anatomical axis that passed through the medial edge of the medial condyle. At the time of surgery, a locking plate was placed at the lateral side hence the lateral end of the lateral condyle was difficult to detect. So, the distance between the medial end of the medial condyle and the cross point to the mechanical axis was measured on the proximal tibia (Fig. 3). Epiphysis-width discrepancy (EWD) was calculated by comparing the width of the affected side and the width of the unaffected side on radiographs at the latest examination. The epiphysis growth rate was calculated by comparing the width at the latest examination with the width immediately before surgery. To analyse the collapse of the residual epiphysis, the anatomical lateral distal femoral angle (aLDFA) for the distal femoral tumors and medial proximal tibial angle (MPTA) for the proximal tibial tumors at the latest examination were compared with those after surgery (ΔaLDFA/MPTA) [20]. Limb-length discrepancy (LLD) was measured at the latest follow-up. All parameters were measured by using computer software (EV Insite Version 3.1.1.218; PSP Corporation, Tokyo, Japan), which was performed independently by two assessors (AT and TH) blinded to patient information and the assessment was duplicated. Status of the epiphyseal plate at the latest examination, the Musculoskeletal Tumor Society (MSTS) scores [21], complications and oncological statuses were also recorded.

## Results

Epiphyseal preservation surgery was performed in 18 patients. Six patients were excluded; five were older than 15 years and the other one patient was excluded because the frozen autograft was retrieved at 6 months after surgery due to the deep infection. Twelve patients met our inclusion criteria. The demographics and results are shown in Table 1. The mean patient age was 11 years (range, 6 to 14 years). The mean follow-up period was 61 months (range, 32 to 90 months). Eight tumors were located in the distal femur, and four were in the proximal tibia. Among the 12 patients, 11 (91.7%) were Enneking stage IIB and 1 (8.3%) was Enneking stage III with resectable lung metastases at the time of diagnosis.

**Table 1:** Dempgrapic data and outcomes

Patient No. /Sex /Age, y	Site	Enneking stage	Type of tumor excision	Freezing method	Graft length, cm	Bone graft	Union time, mo	Epiphysis growth, %		EWD	LLD, mm	Δ aLDFA /MPTA	Epiphyseal plate at laltest examination	MSTS score	Complications /Treatment (mos)	Follow-up, mo	Oncological Outcome
								Affected side	Cotralateral side								
1/F/8	DF	IIB	Trans-metaphyseal	Pedicule	25	VFG	NA	12	13	3	43	4	close	18	Fracture/ORIF (9)	90	CDF
2/F/13	DF	III	Trans-metaphyseal	Pedicule	29	no	NA	3	4	1	18	−2	close	30	Fracture/ ORIF (21)	76	CDF
3/M/11	DF	IIB	Trans-epiphyseal	Free	14	no	35	18	18	−2	38	1	open	30	LLD/ Lenghning (54)	68	CDF
4/M/10	DF	IIB	Trans-physeal	Free	10	no	7	28	29	0	48	−1	open	30	LLD/ Lenghning (53)	64	CDF
5/F/6	DF	IIB	Trans-metaphyseal	Free	17	no	4	22	21	0	42	−1	open	30		48	CDF
6/F/14	DF	IIB	Trans-physeal	Free	8	cancellous allograft	6	7	7	0	1	2	close	27		51	CDF
7/F/9	DF	IIB	Trans-epiphyseal	Free	14	no	3	8	8	1	34	3	open	27	Fracture/ ORIF (35)	48	CDF
8/M/13	DF	IIB	Trans-physeal	Free	14	no	10	4	4	0	24	0	close	27	LR/endoprosthesis replacement (20)	42	NED
9/F/11	PT	IIB	Trans-physeal	Free	8	cancellous allograft	4	13	15	1	17	4	close	30	Infection/ irrigation (44), Fracture/ Cast (68)	90	CDF
10/F/12	PT	IIB	Trans-epiphyseal	Pedicule	11	no	7	9	8	0	6	−1	close	30		90	CDF
11/F/11	PT	IIB	Trans-epiphyseal	Pedicule	8	no	6	6	6	−2	33	−1	open	30		51	CDF
12/F/10	PT	IIB	Trans-epiphyseal	Pedicule	12	no	5	22	20	0	9	0	open	30		41	CDF

Abbreviations: *DF* distal femur, *PT* proximal tibia, *VFG* vascularized fibular graft, *NA* not applicable, *EWD* Epiphysys width discrepancy, *aLDFA* anatomical lateral distal femoral angle, *MPTA* medial proximal Tibial angle, *MSTS* musculoskeletal tumor society, *ORIF* open reduction and internal fixation, *LLD* limb length discrepancy, *LR* local recurrence, *CDF* Continous disease free, *NED* no evidence of disease

**Fig. 4:**
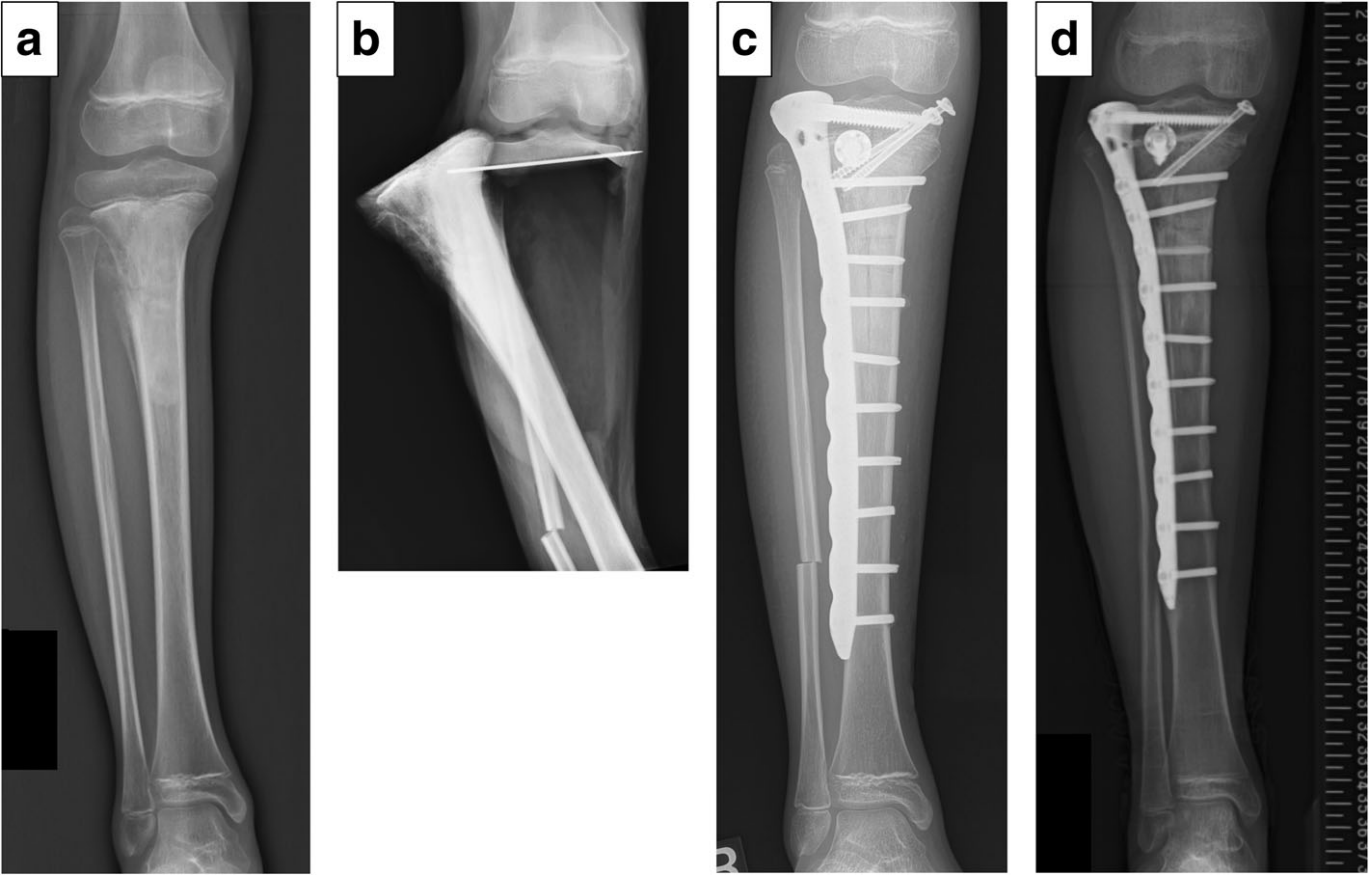
Patient No. 13. A 10-year old girl with osteosarcoma of the right proximal tibia. Preoperative plain radiograph (epiphysis width: 28.3 mm) (**a**). A radiograph after transepiphyseal excision without osteotomy of the distal tibia for the pedicle freezing. A 1.6-mm Kirschner wire was inserted as a guide, positioned under fluoroscopy. Osteotomy of the distal fibula was performed to expose the proximal part of the tibia (**b**). A radiograph after the reconstruction using a tumor-bearing frozen autograft stabilized with locking plate (aLDFA: 87°) (**c**). Whole-leg radiograph after 37 months (aLDFA: 86°, epiphysis width: 33.9 mm) (**d**)

Five (41.7%) patients underwent reconstruction using the pedicle-freezing technique, with 2 for distal femoral tumors and 3 for proximal tibial tumors (Fig. 4). A vascularised fibular graft was used in one patient, while two patients underwent cancellous allograft over the osteotomy site. Tumor excision was transmetaphyseal for 3 distal femoral cases, transphyseal for 3 distal femoral cases and 1 proximal tibial cases, and transepiphyseal for 2 distal femoral and 3 proximal tibial cases. The mean length of tumor-devitalised autograft treated with liquid nitrogen was 14.4 cm (range, 8 to 29 cm), and the mean graft union time was 9 months (range, 3 to 35 months). The mean epiphysis growth was 12.6% (range, 3.3 to 28.0%) of affected side and 12.7% (range, 3.8 to 28.9%) of contralateral side, and EWD was 0.1 mm (range, − 1.0 to 1.7 mm). The mean ΔaLDFA/MPTA was 0.6° (range, − 2.4 to 4.0°). These results indicated that the epiphysis transverse growth was preserved without residual epiphyseal collapse, and the preservation or sacrifice of the epiphyseal plate did not influence the epiphysis transverse growth. The mean limb shortening was 26 mm (range, 1 to 48 mm).

**Fig. 5:**
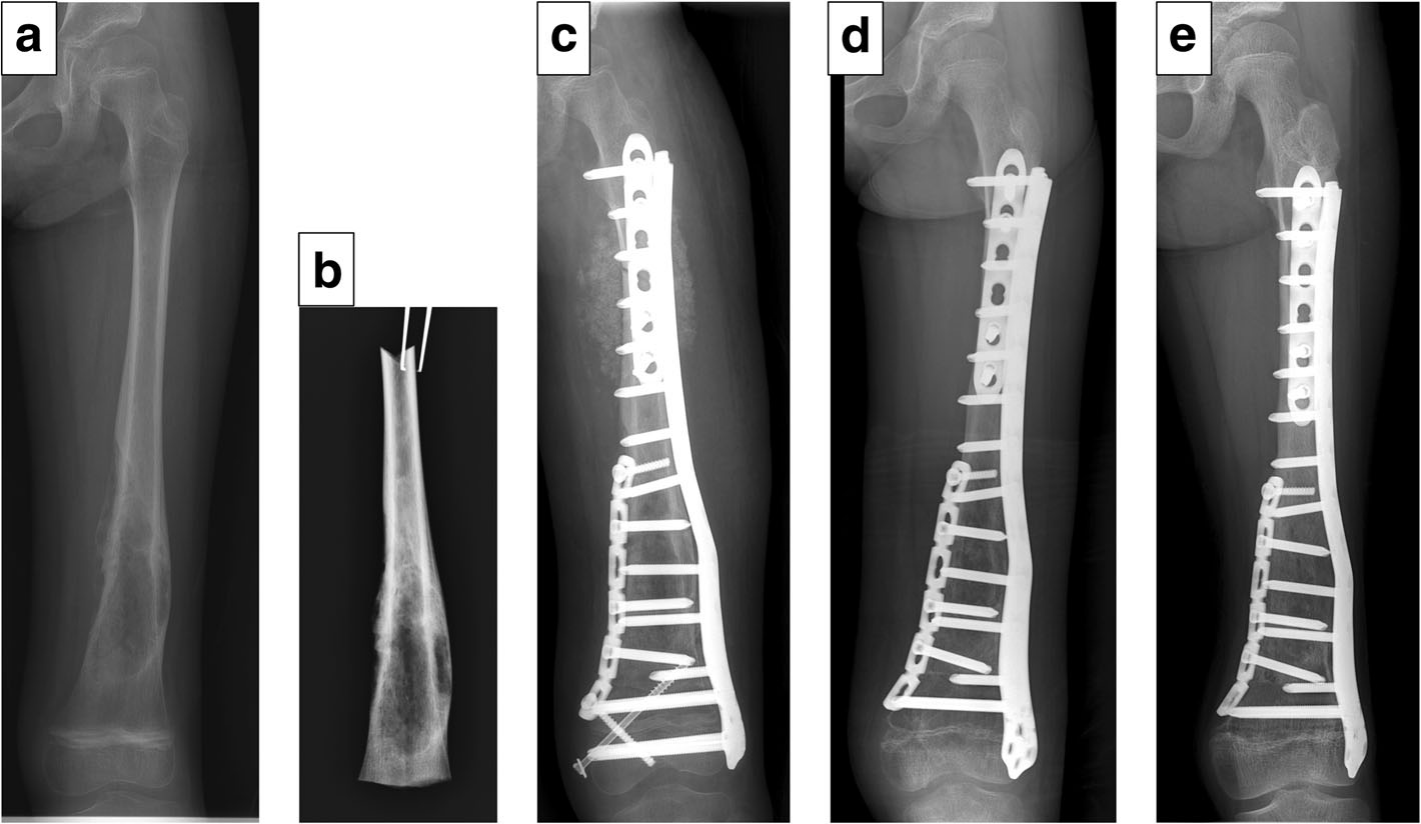
Patient No. 5. A 6-year-old girl with osteosarcoma of the left distal femur (transmetaphyseal excision). Preoperative plain radiograph (epiphysis width: 53.2 mm) (**a**). A radiograph of resected tumor-bearing segment of femur after transmetaphyseal excision (**b**). A radiograph after the reconstruction using a tumor-bearing frozen autograft stabilized with triple-locking plates and a cancellous allograft (aLDFA: 80°) (**c**). A radiograph after 18 months. Screws in the epiphysis were removed (**d**). A radiograph taken 48 months. Latitudinal and longitudinal growth were observed (aLDFA: 83°, epiphysis width: 63.4 mm) (**e**)

Complications that needed additional surgery were observed in 8 (61.5%) patients, that included one deep infection at 6 months after surgery requiring removal of the frozen autograft, and three (25%) fractures. Two of the fracture complications followed pedicle-freezing (Patient No. 1 and 2), and these occurred at the border between the frozen and host bone. One fracture occurred in the frozen bone and was accompanied by plate breakage (Patient No. 7). All three fractures were treated with open reduction and internal fixation combined with allograft or iliac autograft. One local recurrence (8.3%) developed in the surrounding soft tissue in a patient with pathological fracture at the initial presentation, and was treated with re-wide excision including the frozen autograft and reconstruction using an endoprosthesis (Patient No. 8). One superficial infection (8.3%) was managed by irrigation and administration of antibiotics (Patient No. 9). Limb shortening (> 3 cm) was observed in six patients, and two patients (16.7%) (distal femur, 38 mm and 48 mm) underwent limb lengthening of their tibia with three-dimensional external fixation (Taylor Spatial Frame, *Smith and Nephew*, Memphis, USA) (Patient No. 3 and 4). Four cases were managed with shoe lifts. One patient with diaphyseal osteosarcoma underwent initial stabilization of the epiphysis. The screws were removed from the epiphysis after 1.5 years, with subsequent longitudinal growth of epiphysis was observed (Patient No. 5, Fig. 5). The mean MSTS score was 28 (range, 18 to 30). Oncological outcomes were continuous disease free (CDF) in 11 patients and no evidence of disease (NED) in 1 patient at 41 to 90 months follow-up (mean 63 months).

## Discussion

In the present study, we found that the transverse growth of the residual epiphysis was observed with almost same rate with contralateral side (12.6% of affected side and 12.7% of contralateral side), and no apparent discrepancy in epiphysis widths was detected even though the epiphyseal plate was totally sacrificed.

Epiphyseal-preservation surgery for metaphyseal osteosarcoma has been reported by several authors, making use of allograft [8], distraction osteogenesis [9], tumor-devitalised autograft [10], vascularised fibular graft [11], and custom-made implants [6] for reconstruction. Muscolo L et al. reported the thirteen cases with a high-grade metaphyseal osteosarcoma of the knee who had been treated with a transepiphyseal resection (partial epiphyseal preservation) and reconstructed with allograft. One patient died of bone and pulmonary metastases with no evidence of local recurrence, while one patient had a soft tissue recurrence with no recurrence in the residual epiphysis [22]. Their group (Aponte-Tinao L et al.) later reported a large series (35 cases) of osteosarcoma of the knee treated with epiphyseal preservation and allograft reconstruction. The mean MSTS score was 26 at final follow up, and no recurrence in residual epiphyses were described. However, a second surgical procedure was performed in 19 patients (54.3%), three to treat oncologic complications (three local recurrences) and 16 to treat orthopaedic complications, including 11 fractures, three diaphyseal nonunions, and two deep infections. The epiphysis was eventually removed in 5 patients (14.3%) because of fracture (3 patients), amputation (1 patient) or infection (1e patient). Their study included 12 paediatric patients (< 15 years old); however, nothing was mentioned about the growth of the residual epiphysis [8]. In another study, Henderson ER et al. reported thirty-eight patients who were performed lower-limb preservation with an expandable endoprosthesis after bone tumor resection including replacement of distal femur in twenty-five and proximal tibia in five patients. Limb length discrepancy was managed by the lengthening of prosthesis (mean, 4.5 cm) and the mean limb-length discrepancy was 0.7 cm at the latest examination. However, sixteen patients (42%) experienced one or more complications: superficial infection without sequelae in five, soft-tissue failures in four, aseptic loosening in four, structural failure in two and failure due to infection in three cases at a mean of twenty-eight months. Ten patients (26%) required prosthesis revision, and two patients required amputation. The mean MSTS score was 26.4 in distal femoral and 26.7 in proximal tibial replacement [23]. Complications in the present study was occurred in eight of thirteen patients (61.5%), however two limb-shortening which needed the additional lengthening was inevitable. Therefore, the authors considered that the incidence of remaining 6 complications (46.2%) including 3 fractures, 1 soft tissue recurrence, 1 superficial infection and 1 deep infection were comparable with those of allograft (54.3%) and expandable prosthesis (42%). Limb reconstruction using tumor-devitalised autograft treated with liquid nitrogen procedure was introduced in 1999 [10], with advantages reported as follows: simplicity, low cost, preservation of osteoinductive [24] and osteoconductive properties, perfect fit between graft and host bone, sufficient biomechanical strength, avoidance of disease transmission, avoidance of immunological rejection, nondependent of bone bank, nondependent of special equipment and strict thermal control, easy attachment of tendons and ligaments to bone, preserving of bone morphogenetic proteins (BMP) [13]. The pedicle freezing technic has the following additional reported advantages as well: shorter operating time, maintaining of joint continuity in selected patients, decreased osteotomy sites, and a lower rate of graft healing complications. The impossibility of histological analysis of the whole specimen (tumor necrosis analysis) and complications similar to those related to allograft implantation are noted disadvantages. Moreover, because a frozen autograft is a tumor-devitalised bone, the strength of the autograft depends on the integrity of the remaining structures following cryogenic sterilization. For this reason, prerequisites for our frozen autograft methods are an osteoblastic lesion and that massive osteolytic destruction has not taken place [18]. Although Yamamoto N, et al. reported that the compression strength of frozen autograft immediately after the treatment was similar to intact bone in animal model [25], the biomechanical strength will be reduced by devitalized tissue with time.

Wong KC et al. reported eight cases of joint-preserving tumor resection in which image-guided computer navigation and reconstruction using a computer-aided design (CAD) prosthesis were performed with excellent limb function (mean MSTS score, 29). They presented a case of continuous growth of the remaining distal femur epiphysis [6]. Puhaindran ME et al. reported nine cases of paediatric osteosarcoma treated by epiphysis preserving tumor excision and reconstructed with fibula grafts supplemented by autoclaved bone grafts infused with bone marrow. They showed one case of latitudinal residual epiphysis growth following distal femur osteosarcoma excision and reconstruction [26]. The epiphysis in a child is composed of articular cartilage, epiphyseal cartilage, a secondary centre of ossification and the physis, all of which are referred to as the articular-epiphyseal cartilage complex [27]. The secondary centre of ossification is supplied by the epiphyseal artery, branches of which end in the proliferating cartilage zone [28]. The groove of Ranvier and the perichondrial ring of LaCroix, both of which support latitudinal growth of the physis, surround the latter. Although the latitudinal growth arrest after damage to the groove of Ranvier and the perichondrial ring of LaCroix caused by physeal injury has been well discussed [29], the latitudinal growth after epiphyseal-preservation surgery has not been fully clarified.

The authors stabilized the residual small epiphysis with a locking plate for preventing loosening or collapse of the epiphysis. Mei J et al. reported their successful treatment of a pediatric distal femoral osteosarcoma with epiphyseal-preservation surgery using a locking plate. They preserved the epiphyseal plate with temporary screw fixation, and removed the screw after 14 weeks. Subsequently, the distal end of the plate had moved superior to the epiphysis accompanied by bone growth [7]. In the present study, one case showed the longitudinal growth of the epiphysis following removal of the screws from the epiphysis (Fig. 5). Despite temporarily stabilization of the epiphysis across the epiphyseal plate after transmetaphyseal excision, longitudinal growth can be expected following removal of the epiphyseal screw. Limb shortening was a major problem, but two cases of distal femoral reconstruction in the present study were successfully salvaged by distraction osteogenesis of the tibia (Patients No. 3 and 4). Four cases were managed with shoe lifts. In lieu of expandable prostheses, excessive limb shortening shall require additional salvage surgery such as distraction osteogenesis. However, we believe our biological reconstruction has several benefits that help preserve excellent limb function as shown by the MSTS score.

The long-term safety of close surgical margin resection including epiphyseal preservation surgery in the treatment of osteosarcoma is yet to be clarified. The most important consideration is to evaluate tumor extension to the epiphysis accurately. Jesus-Garcia R, et al. reported the epiphyseal plate invasion was detected 44% radiologically in their study, whereas histological examination detected 84%. However, the epiphyseal involvement was evaluated by using the routine radiographs [30]. Hoffer FA et al. reported that epiphyseal extension of an osteosarcoma could be detected on MRI as a dark signal on T1 and a bright signal on STIR in MRI. [5] These findings have led to a consensus that epiphyseal-preservation surgery can be performed [11]. Before this consensus, a few studies have reported use of epiphyseal-preservation surgery, including physeal distraction, when removing the tumor [31]. Andreou D et al. reported that the close margins did not lead to increased local recurrence in their 123 of 1355 cases of osteosarcoma treated with close surgical margin resection [32]. In the present study, our method was indicated for those patients who responded to neoadjuvant chemotherapy and with no tumor extension to the epiphysis. All patients were alive within the mean follow-up period of 61 months (range, 32 to 93 months).,However one (8.3%) of 12 patients underwent removal of the residual epiphysis due to soft tissue recurrence at 20 months after the initial surgery. Careful assessment of tumor extension to the epiphysis by using MRI and more long-term analyses are mandatory to evaluate the safety of this method.

This study had some limitations. The number of patients was small; the patient age at the time of surgery, type of excision used for preservation of the epiphysis and the follow-up term varied and the follow-up periods were relatively short. The growth of residual epiphysis was evaluated by measuring the two-dimensional latitudinal growth only by using plain radiography. Moreover, the epiphysis of the proximal tibia was investigated only by examining half of the entire epiphysis width due to the shadow of the plate. Although Computed tomography (CT) scan is more accurate, we did not routinely preform CT scan to reduce radiation exposures. We did not analyse the longitudinal growth of the residual epiphysis because this study contained three types of osteotomy, and it was also difficult to measure the longitudinal growth after achieving bony union with a transepiphyseal excision. Despite its limitations related to its retrospective nature, the present study provides the possibility of continued residual growth of epiphysis after epiphyseal-preservation surgery in childhood osteosarcomas around the knee. Further prospective studies in a larger series of patients with long-term follow-up should be conducted.

## Conclusion

Epiphysis transverse growth was not diminished, and there was no collapse following epiphyseal-preservation surgery in this limited series of childhood osteosarcoma around the knee. Although careful assessment is necessary to evaluate the extent of tumor involvement of the epiphysis, epiphyseal-preservation surgery has the advantage of preserving adjacent joint function with excellent limb function. Epiphysis-preservation surgery should be considered in children.

## Abbreviations

aLDFA: Anatomical lateral distal femoral angle
EWD: Epiphysis-width discrepancy
LLD: Limb-length discrepancy
MPTA: Medial proximal tibial angle
MRI: Magnetic resonance imaging
MSTS: the Musculoskeletal Tumor Society

## References

[1] Ayerza MA, Farfalli GL, Aponte-Tinao L, Luis Muscolo D (2010). Does increased rate of limb-sparing surgery affect survival in osteosarcoma?. Clin Orthop Relat Res.

[2] Kang S, Lee JS, Park J, Park S-S (2017). Staged lengthening and reconstruction for children with a leg-length discrepancy after excision of an osteosarcoma around the knee. Bone Joint J.

[3] Pala E, Henderson ER, Calabrò T, Angelini A, Abati CN, Trovarelli G (2013). Survival of current production tumor endoprostheses: complications, functional results, and a comparative statistical analysis. J Surg Oncol.

[4] Han C, Chung D, Lee J, Jeong B. Lengthening of intercalary allograft combined with free vascularized fibular graft after reconstruction in pediatric osteosarcoma of femur. J Pediatr Orthop B. 2010:61–5. 10.1097/bpb.0b013e3283330207 19950438

[5] Hoffer FA, Nikanorov AY, Reddick WE, Bodner SM, Xiong X, Jones-Wallace D (2000). Accuracy of MR imaging for detecting epiphyseal extension of osteosarcoma. Pediatr Radiol.

[6] Wong KC, Kumta SM (2013). Joint-preserving tumor resection and reconstruction using image-guided computer navigation tumor. Clin Orthop Relat Res.

[7] Mei J, Ni M, Jia GY, Chen YX, Zhu XZ (2015). Intermittent internal fixation with a locking plate to preserve epiphyseal growth function during limb-salvage surgery in a child with osteosarcoma of the distal femur. Medicine (Baltimore).

[8] Aponte-tinao L, Ayerza MA, Muscolo DL, Farfalli GL. Survival, recurrence, and function after epiphyseal preservation and allograft reconstruction in osteosarcoma of the knee. Clin Orthop Relat Res. 2015:1789–96. 10.1007/s11999-014-4028-5 PMC4385338 25352262

[9] Tsuchiya H, Abdel-Wanis ME, Sakurakichi K, Yamashiro T, Tomita K (2002). Osteosarcoma around the knee. Intraepiphyseal excision and biological reconstruction with distraction osteogenesis. J Bone Joint Surg Br.

[10] Tsuchiya H, Wan SL, Sakayama K, Yamamoto N, Nishida H, Tomita K (2005). Reconstruction using an autograft containing tumour treated by liquid nitrogen. J Bone Joint Surg Br..

[11] Kiss S, Terebessy T, Szöke G, Kiss J, Antal I, Szendröi M (2013). Epiphysis preserving resection of malignant proximal tibial tumours. Int Orthop.

[12] Igarashi K, Yamamoto N, Shirai T, Hayashi K, Nishida H, Kimura H (2014). The long-term outcome following the use of frozen autograft treated with liquid nitrogen in the management of bone and soft-tissue sarcomas. Bone Joint J..

[13] Takata M, Sugimoto N, Yamamoto N, Shirai T, Hayashi K, Nishida H (2011). Activity of bone morphogenetic protein-7 after treatment at various temperatures: freezing vs. pasteurization vs. allograft. Cryobiology.

[14] Tsuchiya H, Tomita K, Mori Y, Asada N, Yamamoto N (1999). Marginal excision for osteosarcoma with caffeine assisted chemotherapy. Clin Orthop Relat Res.

[15] Miwa S, Takeuchi A, Shirai T, Taki J, Yamamoto N, Nishida H, et al. Prognostic value of radiological response to chemotherapy in patients with osteosarcoma. PLoS One. 2013;8 10.1371/journal.pone.0070015 PMC3726455 23922892

[16] Kumta SM, Chow TC, Griffith J, Li CK, Kew J, Leung PC (1999). Classifying the location of osteosarcoma with reference to the epiphyseal plate helps determine the optimal skeletal resection in limb salvage procedures. Arch Orthop Trauma Surg.

[17] Enneking WF, Spanier SS, Goodman MA. A system for the surgical staging of musculoskeletal sarcoma. Clin Orthop Relat Res. 1980;(153):106–20. 7449206

[18] Tsuchiya H, Nishida H, Srisawat P, Shirai T, Hayashi K, Takeuchi A (2010). Pedicle frozen autograft reconstruction in malignant bone tumors. J Orthop Sci.

[19] Freeman TR, Souryal TO (1993). Intercondylar notch size and anterior cruciate ligament injuries in athletes. A prospective study. Am J Sports Med.

[20] Paley D, Herzenberg JE, Tetsworth K, McKie J, Bhave A (1994). Deformity planning for frontal and sagittal plane corrective osteotomies. Orthop Clin North Am.

[21] Enneking WF, Dunham W, Gebhardt MC, Malawar M, Pritchard DJ. A system for the functional evaluation of reconstructive procedures after surgical treatment of tumors of the musculoskeletal system. Clin Orthop Relat Res. 1993;(286):241–6. 8425352

[22] Muscolo DL, Ayerza MA, Aponte-Tinao LA, Ranalletta M. Partial epiphyseal preservation and intercalary allograft reconstruction in high-grade metaphyseal osteosarcoma of the knee. J Bone Joint Surg Am. 2005;87 Suppl 1 (Pt 2):226–36. 10.2106/JBJS.E.00253 16140796

[23] Henderson ER, Pepper AM, Marulanda G, Binitie OT, Cheong D, Letson GD (2012). Outcome of lower-limb preservation with an expandable endoprosthesis after bone tumor resection in children. J Bone Joint Surg Am..

[24] Tanzawa Y, Tsuchiya H, Shirai T, Hayashi K, Yo Z, Tomita K (2009). Histological examination of frozen autograft treated by liquid nitrogen removed after implantation. J Orthop Sci.

[25] Yamamoto N, Tsuchiya H, Tomita K (2003). Effects of liquid nitrogen treatment on the proliferation ofosteosarcoma and the biomechanical properties of normal bone. J Orthop Sci.

[26] Puhaindran ME, Pho RWH (2014). Biological reconstruction for children with osteosarcoma around the knee. Ann Acad Med Singap.

[27] Carlson CS, Hilley HD, Henrikson CK (1985). Ultrastructure of normal epiphyseal cartilage of the articular-epiphyseal cartilage complex in growing swine. Am J Vet Res.

[28] Chung SM (1976). The arterial supply of the developing proximal end of the human femur. J Bone Joint Surg Am.

[29] Peterson HA, Peterson HA (2007). Anatomy and Growth. Epiphyseal growth plate fractures.

[30] Jesus-Garcia R, Seixas MT, Costa SR, Petrilli AS, Filho JL (2000). Epiphyseal plate involvement in osteosarcoma. Clin Orthop Relat Res.

[31] Cañadell J, Forriol F, Cara JA (1994). Removal of metaphyseal bone tumours with preservation of the epiphysis. Physeal distraction before excision. J Bone Joint Surg Br.

[32] Andreou D, Bielack SS, Carrle D, Kevric M, Kotz R, Winkelmann W, et al. The influence of tumor- and treatment-related factors on the development of local recurrence in osteosarcoma after adequate surgery. An analysis of 1355 patients treated on neoadjuvant Cooperative Osteosarcoma Study Group protocols. Ann Oncol. 2011:1228–35. 10.1093/annonc/mdq589 21030381

